# Evolution for the Next Generation

**DOI:** 10.1371/journal.pbio.0020071

**Published:** 2004-03-16

**Authors:** Masakado Kawata

## Abstract

Do you want to know about evolution? Brian and Deborah Charlesworth provide an excellent and concise account of the core issues for a broad range of readers

[Fig pbio-0020071-g001]Evolution is a complex phenomenon that requires a broad understanding of many areas of biology for us to appreciate it fully. Moreover, the field has expanded rapidly, especially since the development of molecular techniques in the past two to three decades. Futuyma's classic text on evolution (1998) contains 26 chapters totaling 763 pages. To cover the topic in only eight chapters and 145 pages, as the Charlesworths have done in *Evolution: A Very Short Introduction*, is no mean feat.

**Figure pbio-0020071-g001:**
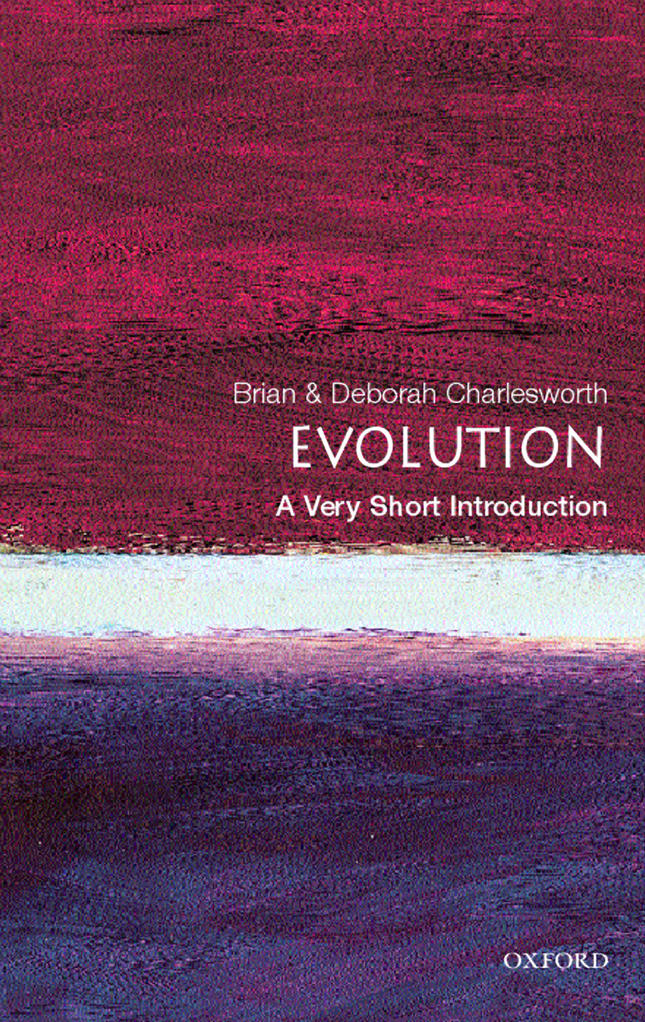


Their book is one of a series of short introductions, published by Oxford University Press, covering an eclectic array of subjects that aim to provide an accessible yet stimulating read for anyone wanting a thorough introduction to a topic. In this small volume, the Charlesworths have succeeded on both fronts and provide an excellent account of the core issues for a broad range of readers. One of the reasons for the book's appeal is that the authors draw on a range of carefully chosen human traits to illustrate their points. By contrast, most evolutionary textbooks (other than those purely on human evolution) tend to focus on nonhuman organisms. As with traits in every other organism, many human and human-related characteristics have evolved via genetic drift and natural selection, and they provide an effective means of convincing readers of the reality and relevance of evolution. For example, to explain how mutation can cause the loss of a function, the authors discuss the relatively poor sense of smell in humans, as compared with many other mammals, using an example of a vestigial ‘pseudogene’ of a human olfactory receptor gene. They also discuss tooth decay, enzyme aesthetics, heritable differences, cancer and other diseases, and the ability to taste and so on.

Although the topics the Charlesworths choose to focus on are certainly appropriate, they provide only a brief mention of one important process—development. Evolutionary developmental biology is a burgeoning field that can provide interesting and important insights into our understanding of the mechanisms of evolution. For example, the absence of eyes in cavefish, rather than being the result of a degenerative process, might be the result of selection on genes that govern feeding morphology, a selection process that has included suppression of eye development (Pennisi 2002). Such developmental mechanisms and constraints can actually alter the direction of evolution. Although the key forces driving evolution are usually thought of as mutation, genetic drift, natural selection, and divergence, the developmental pathways from genes to phenotypes, along with associated developmental constraints, can also determine the rate and direction of evolution.

In Chapter 7, the authors discuss five topics that have traditionally been hard to understand from an evolutionary point of view. These ‘difficult problems’ are ageing, altruism, human consciousness, complex adaptations, and the origin of living cells. Difficult problems can be interpreted in two ways: those that, although hard to solve, have either been explained or will eventually be explained by modern evolutionary theories, and those problems that cannot be fully resolved with our current understanding but leave room for learning about additional mechanisms or factors. The Charlesworths generally consider only those problems of the former type—the explained ones. However, I think that some of the more intractable problems should be described in more detail. For instance, complex adaptation might be fully explained by mutations and natural selection, but additional unknown mechanisms might be essential for the evolution of the complex traits. I realize that opponents of modern evolutionary theory, such as creationists, have often cited these traditional problems to support their conclusion that modern evolutionary theory is wrong; but progress always depends on the consideration of new ideas, and there might be important mechanisms still to be discovered that play a key role in evolution. Describing potentially intractable problems might also spur on young readers who are thinking of studying evolutionary biology with the hope that there are still some theoretical battles to be conquered.

Who is the target audience of this book? For many books, the topics chosen and the writing style can perhaps provide clues to the nature of the readers. For instance, *The Blind Watchmaker* by Richard Dawkins (1990) is a good introductory book for those interested in natural selection because it seems to be written mainly for individuals who either oppose or do not understand the role of natural selection. In the Charlesworths' book, providing evidence for evolution occupies 49 of the 130 pages. They explain how the similarities between living creatures can be understood in terms of evolution (Chapter 3) and subsequently discuss evidence from the geographical distributions of living and fossil species (Chapter 4). My first impression was that this part occupies too large a proportion of the book. However, Chapter 3 serves as a good introduction to the basic background of biology, such as the gene, DNA, and cells. When I read a recent article about a teaching controversy concerning evolution (Scott and Branch 2003), I began to appreciate the importance—at least in the United Kingdom and the United States—of convincing readers of the reality and cogency of evolution and evolution theory by astutely providing them with the evidence to judge for themselves.

In Japan, there seem to be few people who deny the facts of evolution, although there are many ideologically motivated books opposing natural selection and Darwinism. To convince creationists of evolution is usually extremely difficult, if not impossible, because they will never doubt their assumption that God created humankind. Education of young and curious people, however, can make a difference. This is where I think the book will be most successful, but this book should not just be limited to young people—I can recommend it to anyone who wants to know about evolution. Moreover, I can recommend it to Japanese students not only as an introduction to evolution, but also as an exercise in reading a well-written and engaging English text.
